# Impact of new consent procedures on uptake of the schools-based human papillomavirus (HPV) vaccination programme

**DOI:** 10.1093/pubmed/fdaa164

**Published:** 2020-09-26

**Authors:** Harriet Fisher, Matthew Hickman, Joanne Ferrie, Karen Evans, Michael Bell, Julie Yates, Marion Roderick, Rosy Reynolds, John MacLeod, Suzanne Audrey

**Affiliations:** Population Health Sciences, Bristol Medical School, University of Bristol, Bristol, UK; Population Health Sciences, Bristol Medical School, University of Bristol, Bristol, UK; Screening and Immunisations South West, Public Health England, Tauton, UK; Sirona Care & Health, Bristol and South Gloucestershire, Bristol, UK; Bristol Biomedical Research Centre and NIHR CLAHRC West, Bristol, UK; Screening and Immunisations South West, Public Health England, Tauton, UK; Department of Paediatric Immunology & Infectious Disease, Bristol Royal Hospital for Children, Bristol, UK; Population Health Sciences, Bristol Medical School, University of Bristol, Bristol, UK; Population Health Sciences, Bristol Medical School, University of Bristol, Bristol, UK; Population Health Sciences, Bristol Medical School, University of Bristol, Bristol, UK

**Keywords:** HPV vaccination programme, adolescents, consent, policy, quasi experimental study design

## Abstract

**Background:**

Local policy change initiating new consent procedures was introduced during 2017–2018 for the human papillomavirus (HPV) vaccination programme year in two local authorities in the south–west of England. This study aims to assess impact on uptake and inequalities.

**Methods:**

Publicly available aggregate and individual-level routine data were retrieved for the programme years 2015–2016 to 2018–2019. Statistical analyses were undertaken to show: (i) change in uptake in intervention local authorities in comparison to matched local authorities and (ii) change in uptake overall, and by local authority, school type, ethnicity and deprivation.

**Results:**

Aggregate data showed uptake in Local Authority One increased from 76.3% to 82.5% in the post-intervention period (risk difference: 6.2% *P* = 0.17), with a difference-in-differences effect of 11.5% (*P* = 0.03). There was no evidence for a difference-in-differences effect in Local Authority Two (*P* = 0.76). Individual-level data showed overall uptake increased post-intervention (risk difference: +1.1%, *P* = 0.05), and for young women attending school in Local Authority One (risk difference: 2.3%, *P* < 0.01). No strong evidence for change by school category, ethnic group and deprivation was found.

**Conclusion:**

Implementation of new consent procedures can improve and overcome trends for decreasing uptake among matched local authorities. However, no evidence for reduction in inequalities was found.

**Implications and discussion:**

The new consent procedures increased uptake in one of the intervention sites and appeared to overcome trends for decreasing uptake in matched sites. There are issues in relation to the quality of data which require addressing.

## Introduction

The English Human Papillomavirus (HPV) vaccination programme aims to prevent mortality and morbidity from HPV-related diseases, including cervical cancer. Since introduction in 2008, high coverage has been achieved, with over 10 million vaccine-eligible young women receiving immunization against HPV.^1^ Recent evidence highlights the potential for HPV vaccination programmes to eliminate cervical cancer.^2^^,^^3^ Based on emerging evidence for cost-effectiveness, in 2019–2020 the HPV vaccination programme was expanded to include young men aged 12–13 years.

Despite overall high vaccination rates in England, there are wide variations in uptake in different local authorities from 67.8% to 95.3%.^4^ Within the overall uptake rate, there are pockets of significantly lower uptake amongst some populations—such as minority ethnic groups and young people educated in alternative provider settings (e.g. specialist schools for young people with learning disabilities, pupil referral units).^5^ These inequalities in uptake have the potential to widen pre-existing disparities in the incidence of HPV-related cancers by ethnicity.^6^

Barriers to uptake of the English HPV vaccination programme are complex, and include sociocultural beliefs and levels of prioritization for young women to be vaccinated.^7^ The requirement for written parental consent has also been shown to be a barrier to vaccination.^8^ In England, the legal framework allows young people to be vaccinated without parental consent provided they are deemed ‘Gillick-competent’.^9^ However, earlier studies show adolescent self-consent was not widely implemented due to local policies and procedures and reluctance from healthcare professionals.^10^^,^^11^

The introduction of local policies which support alternative strategies of obtaining consent, including adolescent self-consent procedures and verbal consent from parents, has the potential to increase uptake of the HPV vaccination programme. The current study was undertaken as a wider programme of research to explore the practicality and acceptability of implementing new consent procedures in two local authorities in the south–west of England.^12^ Specifically, this study examines the impact of the new consent procedures on uptake of the HPV vaccination programme. Findings related to the acceptability of the new consent procedures have been submitted for publication and will be reported shortly.

We use a quasiexperimental study design to:

(i) compare HPV vaccination uptake in the intervention sites with other sites in England where no such intervention took place; and
(ii) compare uptake of the HPV vaccination programme pre- and post-implementation of the consent procedures in the intervention sites overall, and by local authority, school type, ethnic group and deprivation quintile.

## Methods

### Intervention sites

In response to concerns about low uptake rates in the intervention sites, staff at PHE (South West) developed a ‘South West Template Pathway on Self Consent for School Aged Immunisations’. The aim is to support provider organizations in implementing a self-consent process to support young people to easily access vaccines, support immunizers to feel confident about self-consent and to improve the uptake of immunizations. This policy change was introduced in the two local authorities (comprising the intervention sites) in the south–west of England in the 2016–2017 HPV vaccination programme.

### The intervention

The published study protocol provides a detailed description of the new consent procedures for vaccination in the school-setting.^12^ The study was undertaken when the English vaccination programme was delivered routinely to young women only. In brief, prior to the intervention, young women would only receive the HPV vaccine in the school-setting if a written, affirmative parental consent form had been returned to the immunization team and the young woman provided her assent. Policies for consent pathways are locally determined by each provider and are not consistent leading to different interpretation of guidelines and variation across the country. Under the new procedures, where written parental consent is not received the immunization team make telephone calls to seek parental verbal consent during the vaccination session. Additionally, if parents cannot be contacted during the vaccination session, young women considered ‘Gillick-competent’ by the immunization team can self-consent if they confirm that they have discussed the vaccine with their parents and it would not cause a problem at home if they were vaccinated without written or verbal parental consent. Young women who do not receive the vaccine on the day are provided with written information about community catch-up clinics.

### Change in uptake in intervention sites from comparison local authorities in England

Data reporting for uptake of the English HPV vaccination programme is mandatory.^13^ Data related to the eligible population and the number of doses administered are submitted to Public Health England via ImmForm annually.^14^ These data, aggregated at the local authority level, were retrieved from an openly available data source.^15^ To select local authorities with similar characteristics to the intervention sites for comparison, publicly available secondary school-level data were obtained on ethnicity and eligibility for Free School Meals (a measure of deprivation) during the last 6 years.^16^

Local authorities were assigned to tertiles for the following variables: (i) percentage of young women receiving at least one dose of the HPV vaccine in the 2016–2017 HPV vaccination programme (as it was the most recent data available prior to the change to consent policy); (ii) percentage of secondary school population eligible for Free School Meals (a measure of childhood deprivation); and (iii) percentage of secondary school population classified as White British.

Four local authorities matched identically during the 2016–2017 HPV vaccination programme to Local Authority One in terms of vaccination uptake, Free School Meals, and ethnicity and were selected as comparative local authorities for analysis. The same procedure was followed to match an additional nine local authorities with characteristics corresponding to those for Local Authority Two. The numbers of local authorities matched differed by intervention site due to different underlying population characteristics of Local Authority One and Two.

### Uptake of HPV vaccination programme pre- and post-intervention

Prior to study initiation, permission was sought from Sirona Healthcare and InHealth Intelligence, the organizations with responsibility for the data. The data provided covered the two local authorities that implemented the new consent procedures and covered urban or rural/urban areas in the south–west of England. All records relating to young women eligible for vaccination during the intervention period (born between 1 September 2001 and 31 August 2006) who attended school within the geographical boundaries were retrieved.

In the UK, the Child Health Information System holds demographic and vaccination-related records for each young person registered with a family practice. Using a computerized search, the following data fields were extracted from records: (i) partial date of birth; (ii) postcode; (iii) ethnicity; (iv) dates and location HPV vaccination administered; and (v) name and identifying code of school.

Originally, we planned to seek variables related to receipt of childhood vaccinations as a proxy for parental vaccination beliefs.^5^ Following introduction of the new European Union General Data Protection Regulation (GDPR),^17^ interpretation of information governance legislation by the data providers suggested that this variable could only be provided where individual consent was obtained from general practices identified as the data custodian of this variable—which would be too costly and onerous to obtain—so these data available on Child Health Information System were not extracted for this analysis.

School code was used to assign the local authority responsible for delivery of the HPV vaccine. Partial date of birth was used to allocate the programme year when young women were eligible to receive the HPV vaccine. The following school type categories were applied to each record: (i) comprehensive, non-fee-paying; (ii) private, fee-paying; and (iii) alternative education provider (specialist schools for students with significant additional needs, pupil referral units, young offender units, hospital education service and educated at home).

Individual records were classified as ‘received HPV on time’ if there was a record of at least one HPV vaccine dose administered in the programme year in which the young woman was eligible. Postcodes from individual records were linked to the corresponding lower super output area. A deprivation score was assigned using the Index of Multiple Deprivation 2019^18^ and the sample analysed as quintiles. Ethnicity was grouped as: (i) White British; (ii) Black/Black British; (iii) Asian/British Asian; (iv) Mixed background; (v) Other ethnic group; and (vi) Unknown.

The records of young women were excluded from analyses if the relevant school code was either missing, or invalid (e.g. primary school). In the intervention local authorities, parents are routinely asked antenatally to provide information on ethnicity. Although some parents may refuse to provide this information, missing antenatal ethnicity data may represent young women who were born outside local authority boundaries (e.g. immigrant populations) but are now living and/or attending school in the area. For these reasons, data were not excluded on the basis of unknown ethnicity.

## Analysis

### Change in intervention sites from comparison local authorities in England

The dataset was stratified by pre-intervention (programme years 2015–2016 to 2016–2017) and post-intervention study period (programme years 2017–2018 to 2018–2019). The mean number of young women eligible for vaccination and percentage of young women who received the first dose of the HPV vaccine on time was presented overall for England, and separately for the intervention local authorities and comparison local authorities. To test whether there has been an increase in the uptake of the HPV vaccination after the intervention, the risk difference (difference in two proportions and tests of null hypothesis that there has been no change in uptake) was calculated. The ‘difference in differences’ statistical approach was used to measure differences of uptake of the HPV vaccine between the intervention and comparator groups occurring over time.^19^

### Uptake of HPV vaccination programme pre- and post-intervention

Using individual-level data rather than aggregate data, the risk difference was calculated to test whether there has been an increase in the uptake in the intervention local authorities from the pre-intervention to the post-intervention period. To show whether there was an unintended increase or reduction in health inequalities, risk differences were also calculated to assess whether uptake differed by local authority, school category, ethnic group and deprivation quintile.

Measures of effect are measured as risk differences, difference-in-differences and presented with the corresponding standard errors (SE) and *P*-values. All analyses were performed with Stata statistical package, release 15 (Stata Corp, College Station, TX).

## Results

### Change in intervention sites from comparison local authorities in England

Nationally, the uptake of the HPV vaccine remained stable from the pre-intervention to the post-intervention period (87.1% versus 87.5%, *P* = 0.36). Overall, aggregate data showed uptake in Local Authority One was 76.3% in the pre-intervention period and 82.5% in the post-intervention period (risk difference: 6.2%, *P* = 0.17). In the matched local authorities, change to uptake was from 85.2% to 79.9% in the pre- to post-intervention period (risk difference: −5.3%, *P* = 0.04), giving a difference-in-differences measure of effect as 11.5% (*P* = 0.03). In Local Authority Two, there was no change in uptake from the pre- to the post-intervention period from 84.8% to 87.3% (risk difference: 2.4%, *P* = 0.32). There was no change in the matched local authorities (risk difference: 1.0%, *P* = 0.93), and the overall difference-in-differences measure of effect was 1.5% (*P* = 0.57) (Table 1).

**Table 1:** National uptake of HPV vaccination programme pre- and post-intervention period, by local authorities

	Pre-intervention			Post-intervention				
	Eligible	HPV vaccine dose one uptake		Eligible	HPV vaccine dose one uptake		Risk difference	
	*N*	% (SE)	*P*-value	*N*	% (SE)	*P*-value	% (SE)	*P*-value
England overall	587,610	87.5 (0.3)		620,878	87.1 (0.3)		−0.4 (0.4)	0.36
Intervention site: Local Authority One	2022	Mean (0.1)		2362	Mean (1.7)		+6.2 (1.7)	0.17
Comparator local authorities	1367	85.2 (1.6)		1504	79.9 (1.6)		−5.3 (2.3)	0.04
Difference: Local Authority One-comparator		−8.8 (3.4)	0.02		2.6 (3.4)	0.45	+11.5 (4.8)^*^	0.03
Intervention site: Local Authority Two	1221	84.8 (2.4)		1381	87.3 (0.1)		+2.4 (2.4)	0.49
Comparator local authorities	2733	87.1 (0.6)		2809	88.2 (1.2)		+1.0 (1.3)	0.43
Difference: Local Authority Two-Comparator		−2.3 (2.9)	0.42		−0.9 (2.9)	0.76	+1.5 (3.6)^*^	0.72

Pre-intervention period, 2015–2016 to 2016–2017; post-intervention period, 2017–2018 to 2018–2019; HPV, human papillomavirus; SE, standard error.

^*^Difference-in-differences.

### Uptake of HPV vaccination programme pre- and post-intervention

Records related to 14,501 young women registered with a family practitioner and eligible for routine HPV vaccination during the study period were extracted. Individual records were excluded if the school identifying code was absent or invalid (*n* = 784, 5.4%), the date of birth was invalid (*n* = 63, 0.4%), or partial postcode was missing or invalid (*n* = 364, 2.5%).

Of the 13,290 eligible for vaccination during the study period, the majority attended school in Local Authority One (*n* = 8236, 62.0%), attended comprehensive, non-fee-paying schools (*n* = 11,945, 89.9%), and were classified as White British (*n* = 9085, 68.4%; Table 2).

**Table 2:** Timely HPV vaccination uptake pre- and post-intervention period^*^

		Pre-intervention		Post-intervention			
	Overall	HPV vaccine dose one uptake		HPV vaccine dose one uptake		Risk difference	*P*-value
	*N* (%)	*n*	% (95% CI)	*n*	% (95% CI)	%	
Overall	13,290 (100.0)	6343	87.9 (87.1–88.7)	6947	89.0 (88.3–89.7)	1.1	0.05
Intervention sites							
Local Authority One	8236 (62.0)	3878	85.0 (83.8–86.1)	4358	87.3 (86.3–88.3)	+2.3	<0.01
Local Authority Two	5054 (38.0)	2465	92.5 (91.5–93.6)	2589	91.8 (90.8–92.9)	−0.7	0.34
School category							
Comprehensive, non-fee-paying	11,945 (89.9)	5677	88.9 (88.1–89.8)	6268	89.7 (88.9–90.4)	+0.8	0.17
Private, fee-paying	1042 (7.8)	476	80.5 (79.7–85.9)	556	84.5 (81.5–87.4)	+4.0	0.09
Alternative education providers	303 (2.3)	190	76.8 (70.8–82.8)	113	73.5 (65.3–81.6)	−3.4	0.51
Ethnic group							
White British	9085 (68.4)	4319	92.3 (91.5–93.1)	4766	93.2 (92.5–94.0)	+1.0	0.08
Black/Black British	28 (0.2)	12	58.3 (30.4–86.2)	16	75.0 (53.7–96.2)	16.7	0.35
Asian/British Asian	646 (4.9)	302	89.1 (85.6–92.6)	344	90.1 (87.0–93.3)	+1.0	0.66
Mixed background	24 (0.2)	8	62.5 (29.0–96.0)	16	62.5 (38.8–86.2)	0.0	1.00
Other ethnic group	355 (2.7)	168	85.1 (79.7–90.5)	187	80.7 (75.1–86.4)	−4.4	0.28
Unknown	3152 (23.7)	1534	76.0 (73.9–78.1)	1618	77.6 (75.5–79.6)	+1.6	0.30
Deprivation quintile							
Least Deprived	2646 (19.9)	1298	91.4 (89.8–92.9)	1348	91.6 (90.1–93.1)	−0.2	0.82
2	2632 (19.8)	1253	91.7 (90.2–93.2)	1379	92.6 (91.2–94.0)	+0.9	0.39
3	3666 (20.1)	1263	89.8 (88.1–91.5)	1403	90.7 (89.2–92.3)	0.9	0.41
4	2660 (20.0)	1264	84.0 (82.0–86.0)	1396	86.2 (84.4–88.0)	+2.2	0.12
Most deprived	2686 (20.2)	1264	82.6 (80.5–84.7)	1421	84.0 (82.1–85.9)	+1.4	0.33

^*^Pre-intervention period, 2015–2016 to 2016–2017; Post-intervention period, 2017–2018 to 2018–2019; HPV, human papillomavirus; CI, confidence intervals.

Overall, uptake increased from 87.9% in the pre-intervention period to 89.0% in the post-intervention period (risk difference: 1.1%, *P* = 0.05). There was evidence for an intervention effect for young women attending schools in Local Authority One (risk difference: 2.3%, *P* < 0.01), but no such effect in Local Authority Two (risk difference: −0.7%, 0.34). There was weaker evidence to support an increase in the proportion of young women who received the first dose of the HPV vaccine among those who attended private, fee-paying schools (risk difference: 4.0%, *P* = 0.09) and for those classified as White British (risk difference: 1.0%, *P* = 0.08). Although there was no strong evidence for an intervention effect, the proportion of young women who received the first dose of the HPV vaccine remained substantially lower among those attending alternative education provider settings than the other school categories. For example, in the post-intervention period this was 73.5% (95% CI: 65.3–81.6%) compared to 89.7% (95% CI: 88.9–90.4%) in the comprehensive, non-fee-paying school category. There was no evidence for an effect by deprivation quintile (Table 2).

## Discussion

### Main findings

This study reports the impact of new consent procedures on uptake of the HPV vaccination programme delivered to young women. Analyses on publicly available aggregate data were undertaken to compare trends of uptake in local authorities with similar characteristics. These suggested that the difference-in-differences effect of implementation of the new consent procedures in the local authority with lower uptake was ~12%. Our findings from individual-level dataset obtained from the Child Health Information System suggest there was a small overall increase in the proportion of young women receiving immunization against HPV during the intervention period. There was, however, evidence that uptake increased by ~2% for young women attending schools in Local Authority One.

### What is already known on this topic

The analyses suggest that the implementation of new consent procedures in Local Authority One could be overcoming trends towards lower uptake observed in the matched local authorities. It is currently unknown the reasons for trends in decreasing uptake among these local authorities or why this effect was not observed in Local Authority Two. Despite largely positive public attitudes to vaccination,^20^ recent calls have been made to tackle negative misconceptions of vaccines and limit health misinformation circulating in social media.^21^ It is possible that miscommunication of HPV vaccine messages may have influenced trends in uptake among these local authorities.

Substantially lower uptake of the HPV vaccine by young women in alternative education settings, who may have intellectual disabilities, physical health problems or behavioural issues preventing them from attending mainstream school settings, is a concern. These young women are considered more vulnerable and are already known to experience adverse outcomes across multiple health and social domains. Greater understanding of the barriers to uptake of the HPV vaccination programme by these young women could help improve access and address inequalities in uptake.

### What this study adds

The different effect sizes on data obtained from different data sources suggests the results may be influenced by data quality issues. In the analyses comprising individual-level data, for example, almost 800 (4.5%) records were excluded from analysis on the basis that the school assigned to the record was invalid (e.g. primary school). It is likely that a significant proportion of these young women are no longer resident within the local authority boundaries (e.g. moved abroad or to a different local authority) and would therefore not have been eligible for vaccination by the immunization team.

Despite the denominator of both data sources being similar, recorded uptake of the HPV vaccination differed by data sources. This has several important implications that require addressing. Firstly, publicly available data may not accurately reflect actual uptake. Secondly, organizations responsible for delivery of vaccination programmes may face a loss of income if actual performance and activity is better than the data suggest. Finally, this could negatively impact morale of immunization teams responsible for delivery of the programme.

Demographic growth of vaccine-eligible young women in the intervention local authorities was observed during the intervention period (local authority one: 480; local authority two: 124). During the study period, ~360 additional young women were eligible for the HPV vaccine each year which would impact the capacity of the immunization team to deliver the programme as efficiently. Further, lobby groups targeted school leaders with anti-vaccination messages prior to the 2017–2018 programme being delivered. The impact this had on vaccination decision-making amongst families is unknown.

Recently, Public Health England issued updated guidance for healthcare professionals which supports the use of parent verbal consent and adolescent self-consent as strategies to maximize uptake and reduce catch-up sessions.^22^ An updated data collection tool for the new universal HPV vaccination programme (introduced in 2019–2020) will seek non-mandatory information relating to distribution of consent forms, forms returned, verbal consent and refusals.^23^ This will help provide further evidence on a national basis of the extent to which changes to immunization consent policies can improve young people’s access to, and ultimately uptake of, the HPV vaccination programme. The process evaluation undertaken as part of this study will report the acceptability of different methods of obtaining consent from the perspectives of young women, parents and professionals involved with the HPV vaccination programme.

### Limitations of this study

Our study utilized routinely collected vaccination data eliminating the risk of recall and selection bias. The population-based data relates to vaccinations delivered in school and community settings to all young women registered with a general practice eligible for routine HPV vaccination during the study period. Although the data for this study relates to females only, the findings are also likely to be relevant to the universal HPV vaccination programme as immunization teams’ consent procedures will not differentiate young people by gender.

Some limitations to the study warrant discussion. This study used a quasiexperimental design as a randomized controlled trial was not possible or practical. This is especially relevant for health policies and programmes targeted at the population level.^24^ One of our analyses used a pre- and post-intervention design, without a comparator group. For this reason, we also undertook analyses using national aggregate data to compare uptake in the intervention areas with trends in uptake nationally. We matched local authorities to our intervention sites according to aggregate measures of uptake of the HPV vaccination programme, deprivation and ethnicity. There may be other unmeasured characteristics of the population of the local authorities which could also be influential (e.g. perceptions of adolescent autonomy).

As the study relied on routinely collected information, we did not have access to individual-level measures of socioeconomic status. The study may have been underpowered to detect possible non-trivial differences pre- and post-intervention by ethnicity and school category. We relied on area-based measures of deprivation and therefore our study findings may be subject to ecological fallacy. An issue, common to all routinely collected data, is the possibility of data input errors and missing data. We excluded almost 5% of the data as the information related to school was out-of-date.

## Conclusion

Implementation of new consent procedures appeared to overcome trends for decreasing uptake in one of the intervention sites. However, there was no evidence for an absolute increase or reduction in inequalities in uptake. Data quality issues that appear to underreport uptake should be investigated further. Lower uptake among young women in alternative education settings needs addressing.

## Declarations

### Consent and ethical considerations

Research Ethics Committee approval is not required for evaluation that are designed solely to define or judge current care and measure a current service without reference to a standard. This evaluation involves the retrospective analysis of an anonymised dataset comprising data routinely held on the Child Health Information Services as part of the HPV vaccination programme and does not involve the collection of additional data. Research Ethics Committee approval is therefore not required. However, permission from In Health Intelligence (data custodians) was sought prior to obtaining the dataset.

### LIST OF ABBREVIATIONS

CI: confidence intervals

GDPR: General Data Protection Regulation

HPV: human papillomavirus

LSOA: lower super output area

SE: standard error

### Availability of data and materials

The datasets used and/or analysed during the current study are available from the corresponding author on reasonable request.

Name of the registry: ISRCTN registry. Trial registration number: 49086105 Date of registration: 12 January 2018. URL of trial registry record: www.isrctn.com/ ISRCTN49086105.

## Conflicts of interest

The authors have no conflicts of interest to declare.

## Notes

**Harriet Fisher**, Senior Research Associate

**Matthew Hickman**, Professor in Public Health and Epidemiology

**Joanne Ferrie**, Screening and Immunisations Coordinator

**Karen Evans**, Head of School Nursing and Specialist Nursing Services

**Michael Bell**, Patient and Public Involvement Facilitator

**Julie Yates**, Consultant in Public Health

**Marion Roderick**, Specialist Registrar Paediatric Immunology & Infectious Disease

**Rosy Reynolds**, Senior Research Associate

**John MacLeod**, Professor in Clinical Epidemiology and Primary Care

**Suzanne Audrey**, Senior Research Fellow in Public Health
